# Pilocytic Astrocytoma-Derived Cells in Peripheral Blood: A Case Report

**DOI:** 10.3389/fonc.2021.737730

**Published:** 2021-10-27

**Authors:** Giorgio Volpentesta, Giuseppe Donato, Elisabetta Ferraro, Chiara Mignogna, Riccardo Radaelli, Umberto Sabatini, Domenico La Torre, Natalia Malara

**Affiliations:** ^1^ Department of Medical and Surgical Sciences, University “Magna Græcia”, Catanzaro, Italy; ^2^ Department of Health Sciences, University Magna Græcia, Catanzaro, Italy; ^3^ Department of Biology, University of Pisa, Pisa, Italy; ^4^ Department of Medical and Surgical Sciences, University Magna Græcia, Catanzaro, Italy; ^5^ Department of Experimental and Clinical Medicine, University Magna Græcia, Catanzaro, Italy

**Keywords:** pilocytic astrocytoma, diagnostic liquid biopsy, brain tumors, circulating glial cells, blood brain barrier

## Abstract

Imaging limitations, invasive tissue biopsies and poor information over the course of treatment to evaluate ‘real-time’ tumor dynamics justify the emerging use of liquid biopsies in the field of brain tumors. Circulating tumor cells (CTCs) from high-grade astrocytomas might reach the circulation by crossing the blood–brain barrier. Here, for the first time, CTCs cytology in a case of pylocitic astrocytoma is described. An obstructive hydrocephalous due to a lateral mesencephalic tectum mass occluding the Silvio Aqueduct was diagnosed in a young, 18 years old, male. Considering the location of the tumor and the rapid deterioration of the neurological status, it has been decided to urgency treat the patient with ventriculoperitoneal shunting. Magnetic resonance imaging showed a nodular shaped lesion localized within the left lateral mesencephalic tectum. Stereotactic biopsy was not approachable due significant risk of neurological consequences. The diagnosis was performed by blood sampling, a non-invasive procedure for the patient, in order to provide tumor information. Cytopathological features on detected circulating atypical GFAP positive cells led to pilocytic diagnosis confirmed by the patient’s 68 months outcome.

**Graphical Abstract:**
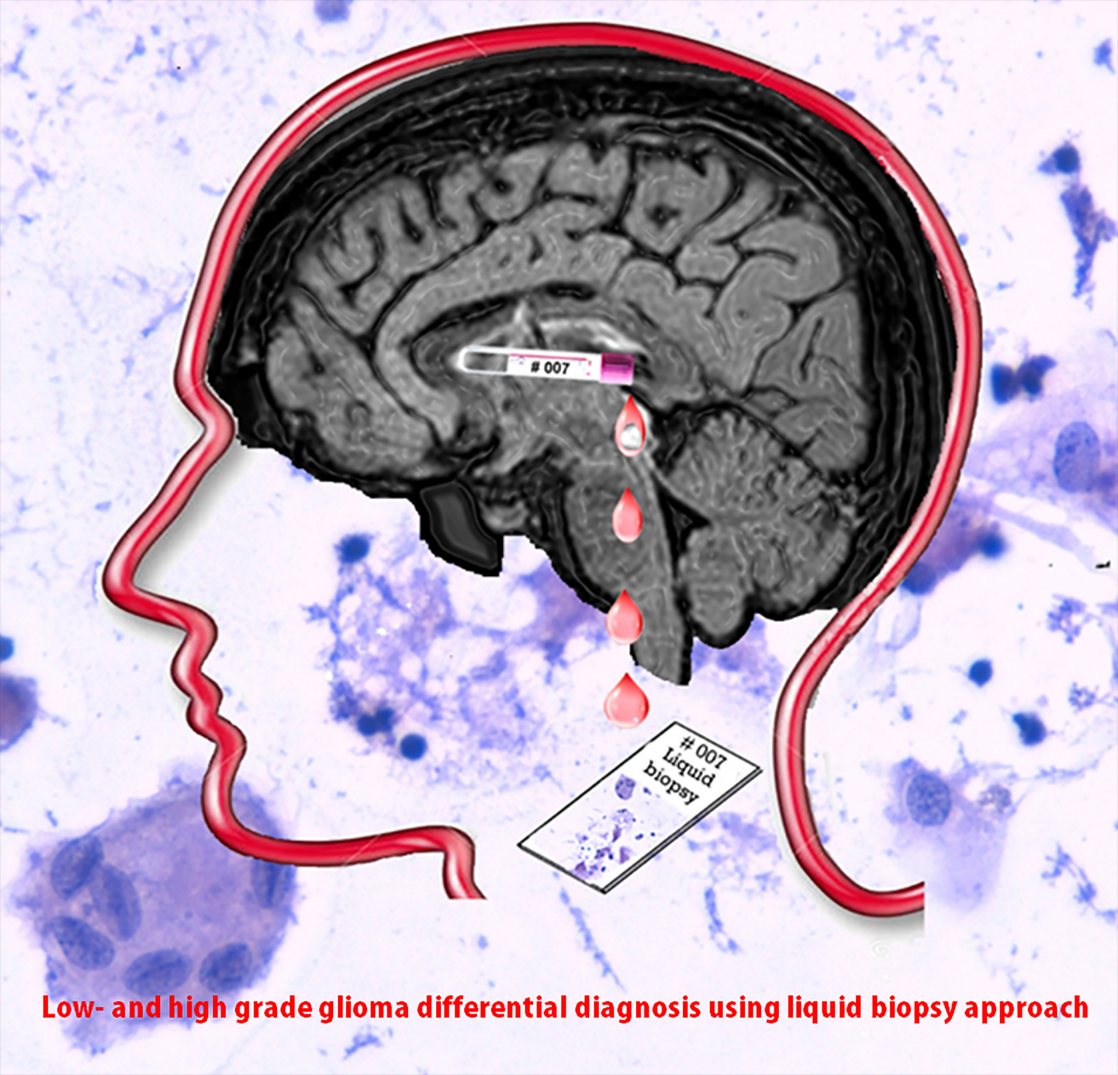


## Introduction

A dramatic improvement in every aspect of intracranial tumors management has occurred in the last two decades. Nevertheless, the combined analysis of clinical signs, symptoms, and magnetic resonance imaging (MRI) and computed tomography (CT)-scan findings is not always diriment to perform a diagnosis, and brain biopsy is required. Many authors have reported significant post-operative complications rate associated to the biopsy procedure (1, 2). A personalized risk assessment for each patient must be evaluated. The negative selection criteria for brain biopsy comprises the lesions located in an eloquent or deep-seated area, like brainstem. As a rule, biopsy is avoided in young adult patient with “diffuse” brainstem glioma (3). Brainstem gliomas account for approximately 10–20% of Central Nervous System (CNS) child tumors. Brainstem gliomas are categorized according to location and appearance using magnetic resonance. They are classified into two types: low grade (or focal/exophytic) and diffuse intrinsic pontine gliomas. Diffuse tumors are typically infiltrating astrocytomas, which can be grade 2–4 depending on histopathological features, and have a poorer outcomes when compared to focal neoplasms (3). In adult tumor’s extension is considered “diffuse” when the lesion is poorly demarcated and is >50% of the brainstem diameter. When the biopsy is performed in the brainstem, a low-grade histology (grade II glioma) is found in up to 80% of cases (4). Occasionally, adult patients can present a rapidly growing tumor similar to the diffuse intrinsic brainstem gliomas, found in paediatric population (4). The classification based on the MRI findings, has been proposed but remains imperfect. Unfortunately, brain stem glioma has an anatomical basis that does not lend itself to easy study. The reason lies in the presence of numerous nuclei of the brain stem essential for the basic functions that alternate with stretches of the white matter. These anatomical features make biopsies and surgery here challenging. These difficulties justify to date also the limited biological knowledge of the pontine lesions of the young adult, compared to those of the child or of the gliobalstoma, identifying them as tumor entities still nosologically in definition.

To increase our knowledge about these tumor entities, characterized by a topographical anatomical dislocation that cannot be reached by conventional biopsy and/or surgical procedures, liquid biopsy represents an alternative and non-invasive strategy. In this field, liquid biopsy is intended as the procedure of isolation and analysis of circulating tumor cells, directly from a single blood sample. We have recently optimized and described an original protocol (5, 6) by which is possible to isolate from a blood sample, and short-time expand, CTCs deriving from different tumor sources included brain primitive neoplasms (7, 8). We have hypothesized that, the short-time expansion of rare circulating non-haematological cells can be useful in order to facilitate the diagnosis, thus avoiding other invasive methods. Therefore, we applied our protocol in this case in which the MRI was unable to identify lesions in the brainstem without a stereotactic biopsy (STB) approach, and the cytological examination of circulating non-haematological cells obtained from the patient blood sample and shortly cultivated led to the final diagnosis- performed by experienced pathologists- of pilocytic astrocytoma.

## Case Presentation

**Figure 1:**
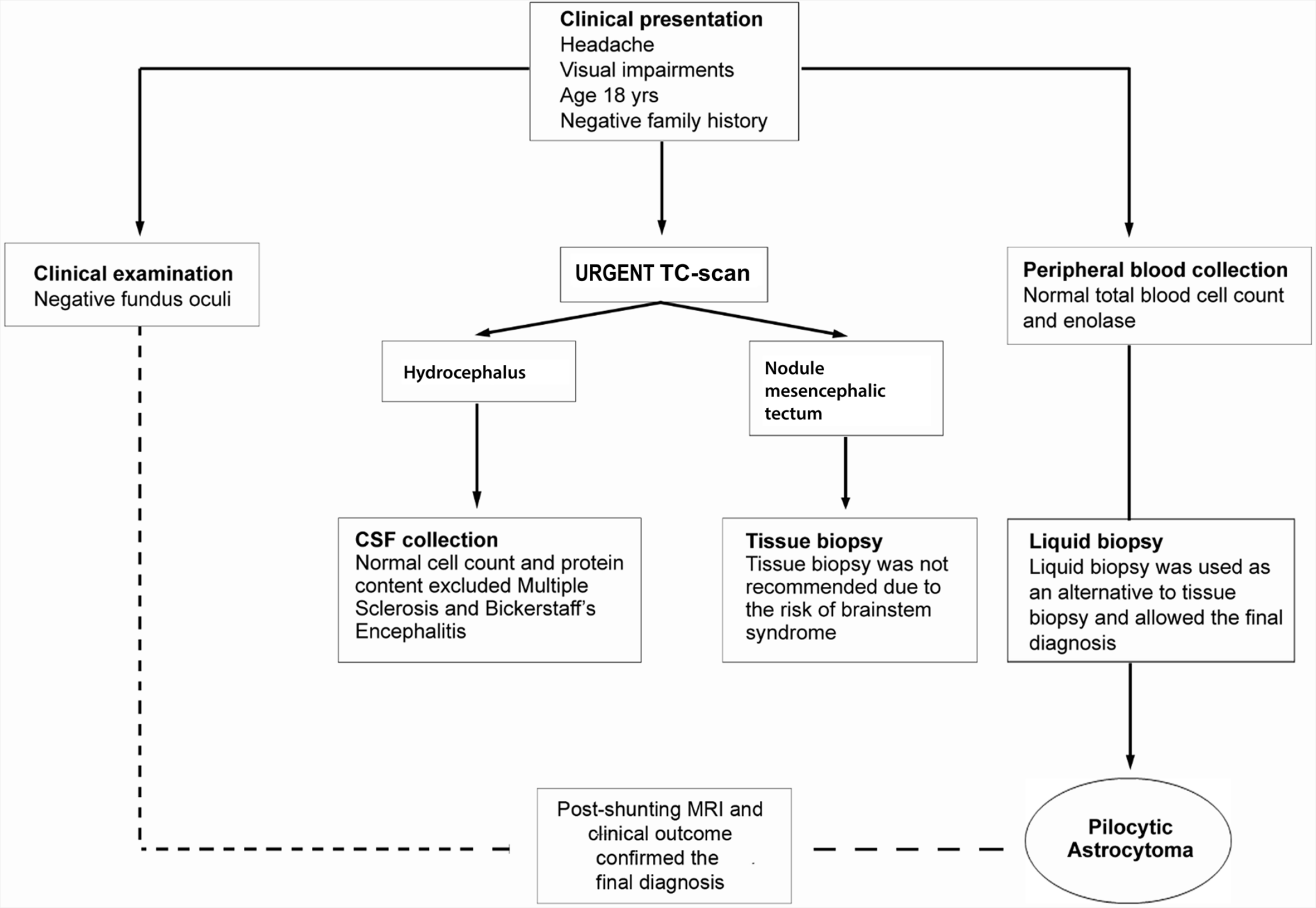
Flow chart of diagnostic work-up. Clinical presentation with headache, visual disturbances, and the intracranial location required differential diagnosis with a wide range of CNS conditions age-related. The patient underwent to clinical examination, instrumental and biochemical analyses. During the hydrocephalus decompression procedure, a sample of CSF fluid was collected. The CSF was clear and the cell count and protein content were normal. Disorders such as multiple sclerosis and Bickerstaff’s encephalitis, conceivable for the clinical symptoms and young age, were excluded. The tissue biopsy needed to exclude high-grade glioma, pineocytoma, germinoma or infectious and parasitic diseases was not recommended. Clinicians opted for performing a protocol of short-time culture on chamber slide to obtain cytological preparations from the blood. Pathological evaluation on liquid biopsy cytological specimens suggested the final diagnosis of pilocytic astrocytoma further confirmed by post-shunting MRI and 68 months of clinical outcome.

A case of a 18-year-old male student having suffered from headache and visual impairments for about two months is reported. Neurological examination was negative. CT-scan performed in urgency revealed the presence of triventricular hydrocephalus caused by stenosis of the cerebral aqueduct. The most urgent therapeutic goal was mainly the management of the hydrocephalus, therefore a ventricular-peritoneal shunt placement (Medos Hakin-programmable valve) was used to provide an alternative flow pathway for the cerebrospinal fluid. The critical location of the lesion discouraged the STB procedure to obtain histopathological specimens available for the diagnosis. In addition, biochemical analysis of cerebrospinal liquor resulted normal with regard to cell count and protein content. Flow chart adopted in the diagnostic phase is reported in **Figure 1**.

**Figure 2:**
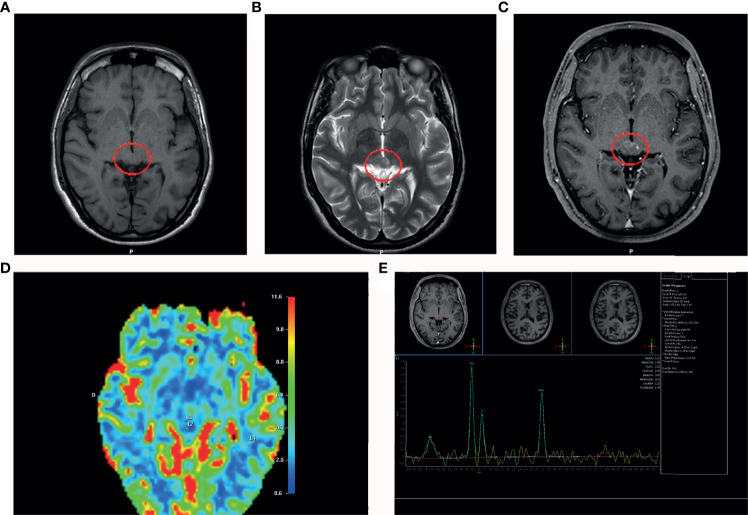
MRI post-shunting (20 days). **(A)** MRI (T1-weighted image) shows a hypointensity signal periaqueductal area (red circle) with irregular profile suggesting the presence of the lesion. **(B)** MRI scan (T2-weighted image) shows a hyperintensity signal in the same area (red circle). **(C)** Gadolinium T1-weighted image shows a low focal contrast enhancement of the lesion in the periaqueductal area. **(D)** Proton magnetic resonance spectroscopy (MRS) reveals elevated cholin peaks (cholin/creatinine ratio at 1,9) in addition to reduced NAA (N-acetylaspartate). **(E)** Perfusion-weighted imaging (PWI) shows a low cerebral blood volume (CBV) in the area of interest.

Post-shunting (20 days) MRI showed a significant decrease of the ventricular size. Moreover, the MRI revealed a nodular lesion within the mesencephalic tectum with hypo-intense areas on the T1-weighted images (**Figure 2A**) and hyper intense signals on the T2-weighted sequence (**Figure 2B**). Gadolinium- T1-weighted image showed a low focal enhancement of the lesion in the periaqueductal area which suggested a reduced damage of the blood-brain barrier (**Figure 2C**). Proton magnetic resonance spectroscopy (H-MRS) revealed elevated choline peaks (choline/creatinine ratio at 1,9) in addition to reduced NAA (N-acetylaspartate) (**Figure 2D**). The perfusion-weighted imaging (PWI) of the lesion showed a low value of relative cerebral blood volume (CBV) (**Figure 2E**). The MR signal alteration was non-specific and could suggest, as alternative diagnostic hypothesis to a neoplasm, inflammatory lesions of various types, demyelinating and vascular.

The impossibility, given the mesencephalic location of the lesion, to perform a STB confirms the importance of the information which could be obtained with the cytological observation of peripheral blood CTCs shortly cultured *in vitro* and obtained applying a protocol previously described (6–8).

**Figure 3:**
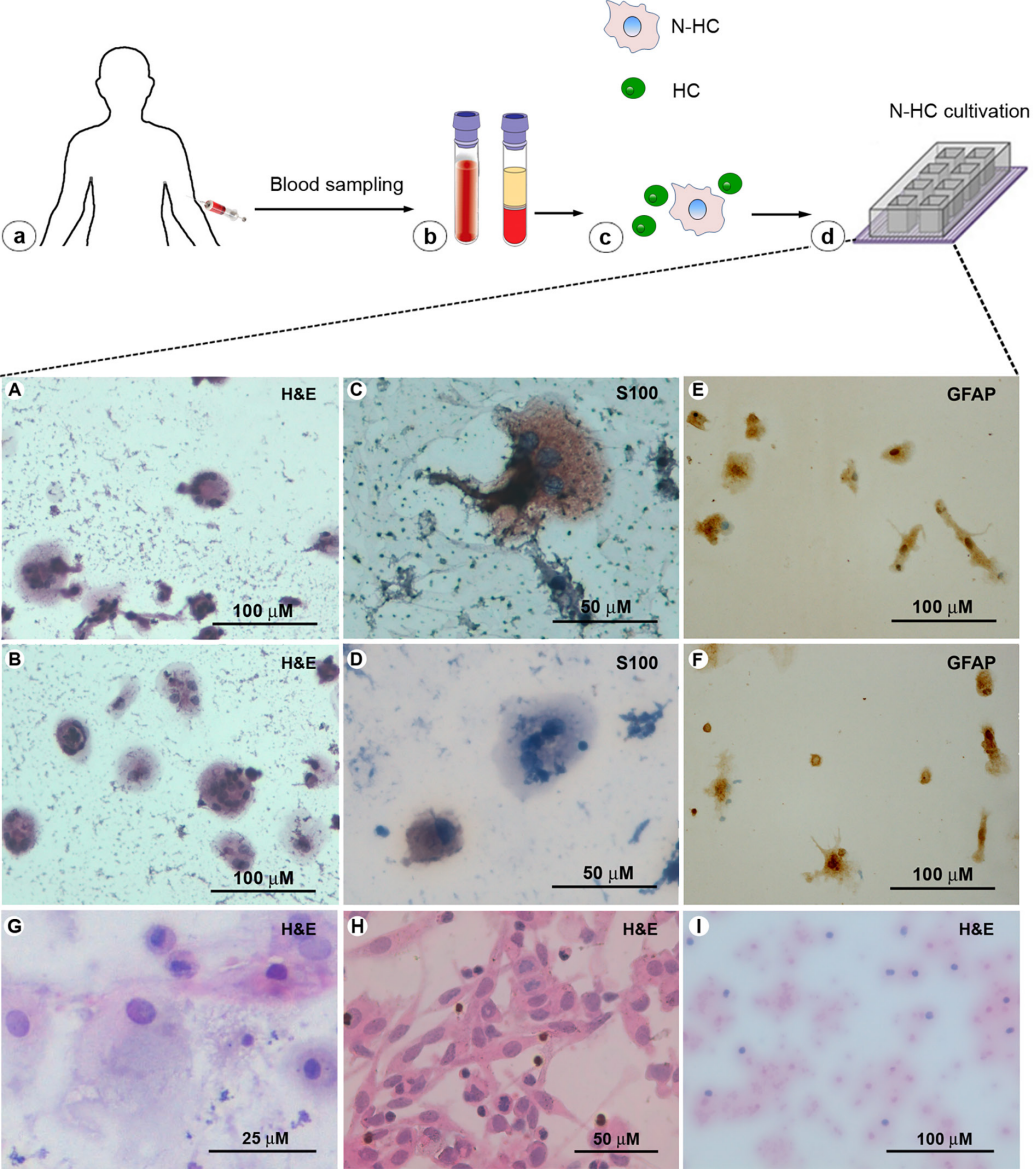
Blood-derived glial cells in cytological preparations. **(a)** Peripheral blood sample collection. **(b)** Blood sample gradient. **(c)** From the gradient, a cellular suspension including non-haematological cells (N-HC) and residual of haematological cells (HC) was isolated. **(d)** The entire cell suspension was seeded in chamber slides and, after 14 days, adherent cells on slide were fixed for cytological analysis. **(A, B)** H&E staining of CTCs deriving from the presented case shows different cellular shapes. **(C, D)** Immunostaining shows CTCs S-100 and **(E, F)** GFAP-positive deriving from the presented case. **(G, H)** Cytological pattern of CTCs derived from one glioblastoma and one cerebral metastasis from melanoma used as positive controls. **(I)** Cytological pattern of cells from a frontal lobe scar used as negative control.

Biomolecular pathways and cell culture plays a pivotal role in cancer research (9–12). However, culture-induced changes in biological properties of tumor cells might profoundly affect research reproducibility and translational potential (13–15). For this reason, the cytological preparations we used in this diagnostic procedure were prepared with a protocol of short-time *in vitro* expansion, which we have widely shown to maintain phenotypic and genotypic features of CTCs (6–9). The volume of the starting blood sample was 5 ml. Briefly, adensity gradient was applied to the blood sample. The cellular suspension isolated in correspondence of the working density phase (**Figures 3a, b**) was seeded on chamber slides (**Figure 3d**) for a short-time expansion of 14 days. The procedure of expansion had two objectives: i) to unmask rare non-haematological cells with atypical proliferation ability *in vitro* and ii) to highlight rare cells numerically sufficient and viable for further characterization. After 14 days, the adherent cells were fixed and stained for cytological examination (13). The culture density of CTCs in the total cultivated cells was of 1:50. The lower rate of atypical cells in the blood-derived culture suggested finding these elements directly in peripheral blood difficult. In fact, the analysis for the presence of negative CD45 cells (CD45 is a marker of the haematological population) was negative in the patient’s whole blood sample.

In this case, as shown in **Figure 3**, the Hematoxylin and Eosin (H&E) stained cytological preparations revealed the presence of bulky, polinucleated or spindled cell atypical elements (**Figures 3A, B**). In addition, these atypical cells found in the blood resulted, at the immunohistochemical analysis performed through methods previously described (14), positive for the expression of the astrocytes markers S-100 (**Figures 3C, D**) and glial fibrillary acidic protein (GFAP) (**Figures 3E, F**) (14, 15). A section of brain human tissue was used as internal positive control. Moreover, as negative control, demonstrating that the reaction visualized is due to the interaction of the epitope of the target molecule and the paratope of the antibody/affinity reagent, parallel assays with the primary antibodies omitted was performed. Moreover, as positive control for the presence of CTCs from cerebral lesions in the peripheral blood we used cytological preparations obtained with the same method from peripheral blood collected (7, 8) from one case of glioblastoma and from one case of brain metastasis from melanoma (cerebral metastasis) (**Figures 3G, H**). As negative control was used the cytological pattern isolated by peripheral blood collected from a case of post-stroke frontal lobe scar (**Figure 3I**). The final cytological diagnosis was of pilocytic astrocytoma because the protean features of the proliferating glial elements (**Figures 3A–F**).

The control MRI (6 months after shunt placement) documented that the lesion was stable. Moreover, the area of altered signal at the quadrigeminal plate, hyperintense on T2/Fluid Attenuation Inversion Recovery (FLAIR), hypointense on T1-weighted images, placed at the entrance of the aqueduct of Sylvius, decreased in width. MRI was repeated evert 6 months in the first 2 years; afterwards, an annual MRI was performed, without changes in the radiological picture. Clinical outcome (68 months) supported by follow up corroborates the pathological diagnosis of pilocytic astrocytoma/low grade glioma.

The patient provided written consent in accordance with ethical principles, relevant guidelines and regulations of the Declaration of Helsinki in accordance with experimental protocols included in the study approved by the local ethical committee, with study number 2013.34. The consent includes also authorization to publish the collected data for medical/scientific purposes in accordance with local laws.

## Discussion and Conclusions

In the present case, the management of hydrocephalus was the most urgent goal, while the management of tectal gliomas, due to the slow growth of this type of tumor, was conservative and included long-term monitoring (16, 17). The monitoring schedule included serial MRI imaging and repeated blood samples for cytological preparations (every six months). Clinicians did not need to prescribe any type of therapy. Clinical outcome (68 months) supported by follow up, confirmed the pathological diagnosis of pilocytic astrocytoma. Successive blood sampling to monitor the number and the pathological detection of cellular elements of interest, reported no significant variation compared to the first pathological evaluation on peripheral blood sample. The patient now is well and leads a normal life.

A major question about our results was: why circulating cells deriving from a benign cerebral lesion can be detectable in the bloodstream? Despite the MRI showed a post-contrast low enhancement of the mesencephalic lesion, this signal supports the hypothesis of the corresponding breached blood-brain barrier (BBB) according to i) the low CBV resulted by PWI findings (**Figure 2E**); ii) low vascularity showed by Gadolinium-enhanced MRI; iii) the evidence of cellular elements brain tissue derived in the bloodstream. We believe that a possible role of the shunt as vehicle of tumor cells in general circulation can be considered unlikely, because it would in turn require an explanation of the entry into the circulation of neoplastic cells at the peritoneal level. The discovery of biomarkers for BBB permeability is starting to happen. The detection of CTCs in the bloodstream could also be an early index of damage of the BBB in brain tumors that precedes in time the neuroradiological signs detectable, for example, through the signal alterations of the MRI. Furthermore, our approach for analyzying circulating tumor elements can provide a more sensitive and integrative method than neuroradiological imaging for the study of barrier damage. These integrated parameters (18) can provide a better diagnostic and prognostic value. However, to date, the identification of ideal peripheral markers to graduate the BBB damage entity remains a challenge for radiologist and neuro-oncologist (19, 20). Our approach suggested that the recognition of circulating cells of brain origin could be useful to unmask the BBB dysfunction. On these considerations, the cytological preparations from peripheral blood could be considered a precious tool also in the diagnosis and monitoring the intracranial lesions, especially in paediatric age, in order to reduce side effect of invasive procedure.

Pyloytic astrocytoma is a slow-growing, circumscribed tumor frequently occurring in children and young adults in cerebellum optic nerves or brain stem. In the brain stem, it may occur as exophytic or deep lesions that may be difficult to biopsy. Through our procedure it was possible to formulate a diagnosis that allowed to decide the patient’s long-term management, avoiding further dangerous or useless diagnostic approaches. The possibility of having in short-term culture a population of cells with characteristics superimposable to single CTCs that may or may not be detected by other methods, may be an opportunity to verify the value of molecular biology techniques such as cancer genome studies and single-cell transcriptome for tumor heterogeneity. Such techniques could definitively prove their usefulness for a personalized approach to patient diagnosis and therapy (21, 22). We believe that this case highlights the strong potential of liquid biopsy to improve the diagnostic and prognostic evaluation and the quality of life in neuro-oncology.

## Data Availability Statement

The raw data supporting the conclusions of this article will be made available by the authors, without undue reservation.

## Ethics Statement

The study was approved by the local ethical committee, with study number 2013.34. The patients/participants provided their written informed consent to participate in this study.

## Author Contributions

Conceptualization, NM and GV. Writing—original draft preparation, NM and GD. Writing—review and editing, NM, GD, and CM. Cytological evaluation: GD and CM. Visualization, NM and RR. Supervision, GD, GV, DT, US, and NM. All authors contributed to the article and approved the submitted version.

## Funding

This work was supported by the “Matteo’s friends Association”.

## Conflict of Interest

The authors declare that the research was conducted in the absence of any commercial or financial relationships that could be construed as a potential conflict of interest.

## Publisher’s Note

All claims expressed in this article are solely those of the authors and do not necessarily represent those of their affiliated organizations, or those of the publisher, the editors and the reviewers. Any product that may be evaluated in this article, or claim that may be made by its manufacturer, is not guaranteed or endorsed by the publisher.

## Abbreviations

MRI, Magnetic resonance imaging; CT, Computed tomography; CTCs, Circulating tumor cells; STB, Stereotactic biopsy; H-MRS, Proton magnetic resonance spectroscopy; NAA, N-acetylaspartate; PWI, Perfusion-weighted imaging; CBV, Cerebral blood volume; CNS, Central nervous system; CSF, Cerebrospinal fluid; FLAIR, Fluid attenuation inversion recovery; H&E, Hematoxylin and eosin; GFAP, Glial fibrillary acidic protein; BBB, Blood-brain-barrier.
